# Intravitreal Dexamethasone Implant in Anti-Vascular Endothelial Growth Factor Pretreated Diabetic Macular Edema—A Swiss Cohort Study

**DOI:** 10.3390/ph17091235

**Published:** 2024-09-19

**Authors:** Ferhat Turgut, Gábor M. Somfai, Christoph Tappeiner, Katja Hatz, Irmela Mantel, Aude Ambresin, Guy Donati, Viviane Guignard, Dana Nagyová, Isabel B. Pfister, Christine Schild, Justus G. Garweg

**Affiliations:** 1 Department of Ophthalmology, Stadtspital Zürich, 8063 Zurich, Switzerland; ferhat.turgut@stadtspital.ch (F.T.);; 2 Spross Research Institute, 8055 Zurich, Switzerland; 3 Department of Ophthalmology, Semmelweis University, 1428 Budapest, Hungary; 4 Gutblick Research, 8808 Pfäffikon, Switzerland; 5 Department of Ophthalmology, Pallas Kliniken, 4600 Olten, Switzerland; 6 Department of Ophthalmology, University Hospital Essen, University Duisburg-Essen, 45147 Essen, Germany; 7 Medical Faculty, University of Bern, 3012 Bern, Switzerland; 8 Vista Augenklinik Binningen, 4102 Binningen, Switzerland; 9 Medical Faculty, University of Basel, 4001 Basel, Switzerland; 10 University Eye Hospital Jules Gonin, 1004 Lausanne, Switzerland; 11 Swiss Visio Montchoisi, 1006 Lausanne, Switzerland; 12 Centre Ophtalmologique de la Colline, Clinique la Colline, 1205 Geneve, Switzerland; 13 Pallas Klinik, 8600 Dübendorf, Switzerland; 14 Swiss Eye Institute and Clinic for Vitreoretinal Diseases, Berner Augenklinik, 3007 Bern, Switzerland; 15 Department of Ophthalmology, Inselspital, University of Bern, 3012 Bern, Switzerland

**Keywords:** diabetic macular edema, intravitreal dexamethasone implant, anti-VEGF

## Abstract

Background/Objectives: Diabetic macular edema (DME) is a significant cause of visual impairment, often treated with anti-vascular endothelial growth factor (anti-VEGF) agents. However, some patients do not respond adequately to this treatment. This study aims to evaluate the contribution of the intravitreal dexamethasone (DEX) implant as a second-line treatment in DME patients with insufficient response to anti-VEGF therapy or with high treatment burden. Methods: This retrospective multicenter cohort study was conducted across seven clinical sites in Switzerland. The study included eyes with active DME that had been pretreated with anti-VEGF for at least six months before receiving DEX therapy. Data were extracted from electronic patient records, focusing on best-corrected visual acuity (BCVA), central subfield thickness (CST), and injection frequency. Results: A total of 95 eyes from 89 patients (38.8% females, mean age 65.6 ± 9.1 years, follow-up time 80.6 ± 38.5 [13.5–166.7] months) were analyzed. Prior to the first DEX implant, eyes had undergone an average of 16.0 ± 13.3 anti-VEGF injections over 32.5 ± 22.4 months. Post-DEX treatment, 22.1% of eyes received DEX monotherapy, 44.2% received a combination of DEX and anti-VEGF, 25.3% continued with anti-VEGF monotherapy, and 8.4% received no further treatment. The number of anti-VEGF injections decreased significantly from 6.4 ± 3.1 in the year before DEX to 1.6 ± 2.4 in the year after DEX (*p* < 0.001). BCVA remained stable (0.4 ± 0.3 logMAR at baseline, 0.4 ± 0.5 logMAR at 24 months, *p* = 0.2), while CST improved from 477.7 ± 141.0 to 320.4 ± 125.5 μm (*p* < 0.001), and the presence of retinal fluid decreased from 98.0% to 61.1% (*p* = 0.021). During follow-up, 26.3% of eyes required glaucoma medication, 4.2% underwent glaucoma surgery, and 1.1% needed cataract surgery. Conclusions: In real-world clinical settings, the addition of DEX to anti-VEGF therapy in DME patients significantly reduces treatment burden and retinal fluid while maintaining visual function. Treatment decisions should balance anatomical and functional outcomes, considering individual patient needs.

## 1. Introduction

Diabetic macular edema (DME) and the corresponding visual impairment pose a significant challenge to the affected patients. Based on their remarkable success in reducing macular edema and improving visual acuity (VA), current therapeutic approaches advocate anti-vascular endothelial growth factor (anti-VEGF) agents as the first-line therapy [1]. However, not all patients achieve sufficient fluid control under anti-VEGF therapy alone due to the complex underlying pathophysiology, including, beyond others, inflammatory pathways [2]. This often results in the need for frequent injections, leading to reduced patient compliance and an increased treatment burden. Failure to control intra- or subretinal fluid and treatment delay will, in the long-term, result in irreversible retinal cell loss and neuroretinal atrophy, along with irreversible visual function loss and a relevant reduction in quality of life [2,3]. In these instances, it seems important to target alternative treatment pathways before irreversible visual loss has been encountered in DME.

In recent years, interest in adjunctive therapies to enhance the outcomes of anti-VEGF therapy in DME patients has grown. Intravitreal dexamethasone, or DEX (Ozurdex®; Allergan, an AbbVie company, North Chicago, IL, USA), implants have been approved as a first- and second-line therapy for DME and gained attention as a promising treatment option due to their potent anti-inflammatory properties and notable drying effects [4]. However, their use is associated with increased rates of secondary glaucoma, cataract formation, and endophthalmitis [4,5]. Given the contribution of inflammatory pathways, the consideration of intravitreal corticosteroids has been recommended at least in patients with DME that does not completely respond to anti-VEGF drugs to improve the treatment response [6,7].

Regarding the potential benefits of DEX, there is a need for additional long-term real-world data on their efficacy and safety when used in combination with anti-VEGF drugs or as monotherapy. Existing studies have yielded varying results, and there is limited long-term data on the outcomes of this combination therapy [8]. DEX, either as monotherapy or in combination with anti-VEGF agents, holds promise as a treatment option for DME in patients who do not achieve sufficient control of retinal fluid under anti-VEGF drugs alone [9]. To the best of our knowledge, three existing studies in Switzerland, with relatively smaller patient cohorts of up to 47 individuals, have investigated the effect of DEX on DME [10,11,12]. Our study seeks to expand on this by providing real-world data from a larger cohort of eight centers in Switzerland.

In this retrospective multicenter cohort study, we aimed to evaluate the real-world outcomes of DEX in Swiss DME patients with insufficient response to anti-VEGF drugs, with a particular focus on its impact on anti-VEGF treatment demand and visual outcomes after the early and late addition of DEX.

## 2. Results

### 2.1. Demographic Data

The study included 95 eyes from 89 patients with DME, 33 (38.8%) of whom were females, with 47 right and 48 left eyes. At the diagnosis of DME, the mean age of the patients was 65.6 ± 9.1 (median 65.3; interquartile range [IQR]: 58.9–70.4) years. At the time of the first DEX, the mean age of the patients was 68.3 ± 9.0 (median 68.6; IQR: 62.7–73.8) years. At the addition of DEX, 27 eyes (28.4%) were phakic, 49 eyes (51.6%) were pseudophakic, cataract surgery in 11 eyes (11.6%) had been performed between the first anti-VEGF injection and first DEX, and for 8 eyes, the information was missing. At the point of initiation of anti-VEGF treatment, six eyes (6.3%) were receiving glaucoma medication, whereas at the first DEX, eight eyes (8.4%) were under glaucoma medication.

**Figure 1:**
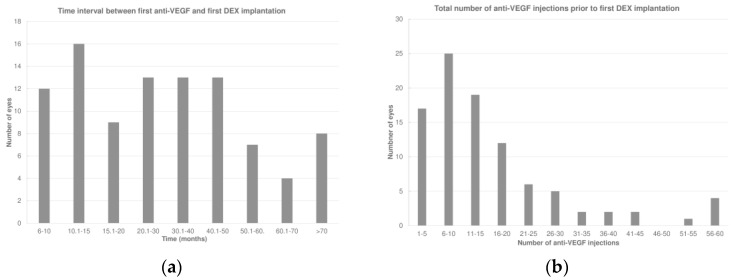
(**a**) Time interval between the first anti-vascular endothelial growth factor (anti-VEGF) and first intravitreal dexamethasone (DEX) implant. (**b**) Total number of anti-VEGF injections prior to the first DEX.

The total duration of follow-up was 80.6 ± 38.5 (median 80.7; IQR: 43.6–103.4) months. The first DEX was performed 32.5 ± 22.4 (median 27.1; IQR: 12.9–44.8) months after the first anti-VEGF therapy (Figure 1a). Prior to receiving DEX, the eyes had undergone 16.0 ± 13.3 (median 13; IQR: 7–19) anti-VEGF injections (Figure 1b). During this period, 47 eyes (49.5%) had received only one type of anti-VEGF treatment, while 48 eyes (50.5%) had undergone a switch to at least another anti-VEGF agent before receiving the first DEX.

**Figure 2:**
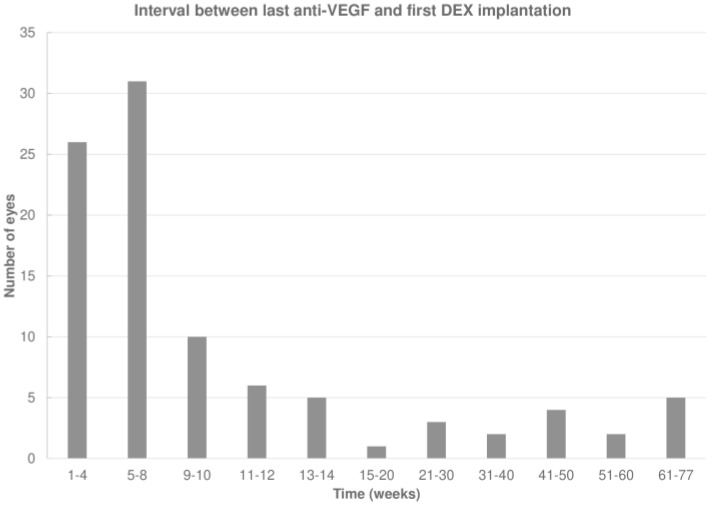
Interval between the last anti-vascular endothelial growth factor (anti-VEGF) and first intravitreal dexamethasone (DEX) implant.

The last anti-VEGF agent administered before DEX was aflibercept, ranibizumab, or an off-label anti-VEGF drug (bevacizumab or ziv-aflibercept) in 54 (56.8%), 39 (41.1%), and 2 (2.1%) eyes, respectively. The mean interval between the last anti-VEGF injection and the first DEX was 13.9 ± 17.4 (median 6.9; IQR: 4.4–12.0) weeks, which increased to 20 ± 14.1 (median 17.0; IQR: 14.0–22.3) weeks after the first DEX (*p* < 0.001; Wilcoxon signed-rank test; Figure 2). 

The decision to administer the first DEX during the anti-VEGF treatment was driven by persistent or recurrent fluid, high treatment demand, and increased treatment burden not supported by the patient in 76 (80%), 12 (12.6%), and 7 (7.4%) eyes, respectively.

### 2.2. Patient Characteristics Following the First DEX

Patients were followed-up for a mean of 48.1 ± 29.7 (median 37.4; IQR: 22.6–69.4) months after the first DEX. After the first DEX, 22.1%, 44.2%, 25.3%, and 8.4% of eyes received a monotherapy with DEX, a combination of DEX and anti-VEGF, a monotherapy with anti-VEGF, or no further treatment, respectively. In the first and second year after the first DEX, a further 2.1 ± 1.3 and 1.3 ± 1.4 DEX were implanted, respectively. In parallel, the number of anti-VEGF injections decreased significantly in the first 12 months after the first DEX (*p* < 0.001; Table 1). 

No further intravitreal injections after the first DEX were reported in eight instances (8.4%). In 24 eyes (25.3%) with ongoing anti-VEGF therapy, a further anti-VEGF injection was required 15.6 ± 10.4 (median 14.3; IQR: 8.8–19.0) weeks after the first DEX, compared to 19.0 ± 6.1 (median 17.7; IQR: 14.9–21.2) weeks for a second DEX in 21 eyes remaining on DEX without ongoing anti-VEGF (22.1%; *p* = 0.021). 

Eyes under DEX monotherapy received 3.0 ± 0.9 (median 3.0; IQR: 2.0–4.0; *n* = 21) DEX within the first 12 months, whereas eyes with combination therapy with anti-VEGF or with DEX and concomitant anti-VEGF received 1.9 ± 1.3 DEX (median 2.0; IQR: 1.0–3.0; *n* = 42), as well as 2.2 ± 2.5 (median 1.5; IQR: 0–4.0; *n* = 24) anti-VEGF injections within the first 12 months after the first DEX (*p* < 0.01). In the second year, eyes under DEX monotherapy received 2.0 ± 1.3 (median 2.0; IQR: 1.0–3.0) DEX, whereas eyes with a combination therapy including anti-VEGF or DEX and concomitant anti-VEGF received 1.2 ± 1.4 DEX (median 1.0; IQR: 0–2.0), as well as 2.8 ± 2.9 (median 2.0; IQR: 0–4.0) anti-VEGF injections (*p* < 0.01).

### 2.3. Change in Visual Acuity over Time 

**Figure 3:**
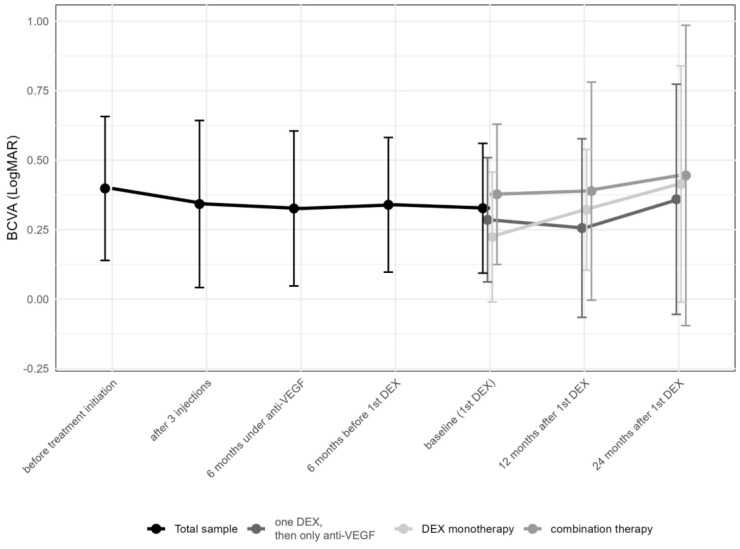
Change in best-corrected visual acuity in logMAR over time from first anti-vascular endothelial growth factor (anti-VEGF) treatment to the 24-month follow-up. Subgroups represent eyes receiving only one intravitreal dexamethasone (DEX) implant (*n* = 24, 25.3%), those undergoing DEX monotherapy during the first year (*n* = 47, 49.5%), and finally, those under combination therapy with DEX and anti-VEGF drugs (*n* = 24, 25.3%). The data points are shown as mean and standard deviation (whiskers).

Best-corrected visual acuity (BCVA) changed from 0.4 ± 0.3 (median 0.3; IQR: 0.2–0.6) logMAR prior to the first anti-VEGF injection to 0.3 ± 0.2 (median 0.3; IQR: 0.2–0.4) logMAR at the time of the first DEX (*p* = 0.49), and remained stable thereafter over 12 to 24 months (0.4 ± 0.3 [median 0.3; IQR: 0.2–0.5] months and 0.4 ± 0.5 [median 0.3; IQR: 0.1–0.5; *p* > 0.05] logMAR, respectively [*p* > 0.05, each]; Figure 3). No significant changes in BCVA were observed either at the time of the first DEX or during the follow-up period (Table 2). 

When comparing eyes with multiple DEX injections with ongoing anti-VEGF injections (*n* = 42), eyes receiving only a single DEX along with ongoing anti-VEGF treatment (*n* = 24) and eyes on a DEX monotherapy (*n* = 21), no differences in BCVA changes were found (Kruskal–Wallis H test, *p* > 0.05). Between the initiation of anti-VEGF treatment and the first DEX, in 31.6% of eyes, BCVA improved by at least two lines, 55.8% maintained vision (±two lines), and in 11.6% of eyes, vision worsened by two lines or more. From the first DEX to 12 months thereafter, 11.6% of eyes improved by at least two lines, 72.6% maintained vision (±two lines), and in 7.4% of eyes, vision worsened by two or more lines (information missing for 8.4% of the data). From the first DEX to 24 months later, 14.7% of eyes improved vision by at least two lines, 49.5% maintained vision (±two lines), and in 9.5% of eyes, vision worsened by two lines or more (information missing for 26.3% of the data).

### 2.4. Central Subfield Thickness and Retinal Fluid

**Figure 4:**
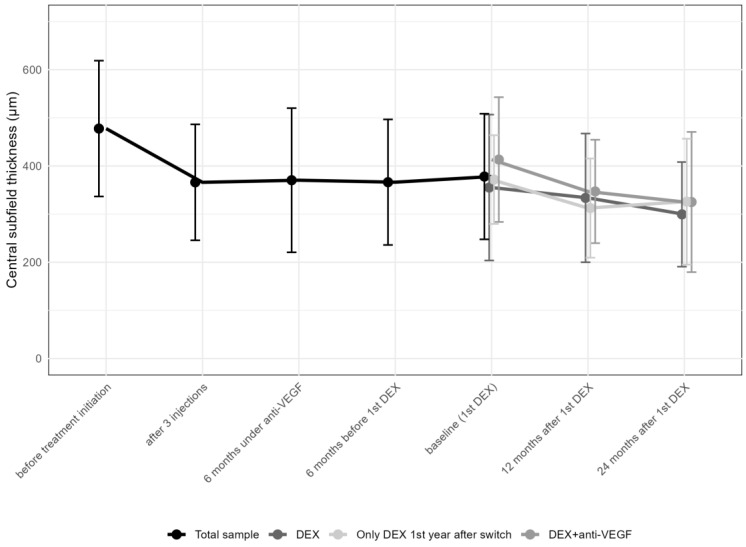
Change in central subfield thickness from the first anti-vascular endothelial growth factor (anti-VEGF) treatment to the 24-month follow-up. DEX: intravitreal dexamethasone implant CST values in the various subgroups, including those receiving multiple DEX injections with anti-VEGF, those with a single DEX plus anti-VEGF, and those on DEX monotherapy, showed comparable results at different time points (Table 4).

Central Subfield Thickness (CST) improved from 477.7 ± 141.0 (median 473.5; IQR: 356.0–591.8) μm at the initiation of anti-VEGF therapy to 366 ± 120.4 (median 337.5; IQR: 272.5–440.3; *p* > 0.05) μm after the first three anti-VEGF injections, and to 378 ± 130.4 (median 354.0; IQR: 290.0–446) μm before the first DEX, improving to 327.3 ± 121.5 (median 299; IQR: 248.0–381.8; *p* < 0.001) μm 12 months after DEX and thereafter remaining stable (320.4 ± 125.5 [median 282.0; IQR: 244.0–363.0]; *p* > 0.05; Table 3 and Figure 4). 

The portion of eyes with persistent fluid decreased from 98% at the time of the addition of DEX to 79% at 12 months (*p* = 0.002) and to 61.1% at 24 months thereafter (*p* = 0.021).

### 2.5. Timing of the First DEX: Early (6–26 Months) vs. Late (>26 Months) 

**Figure 5:**
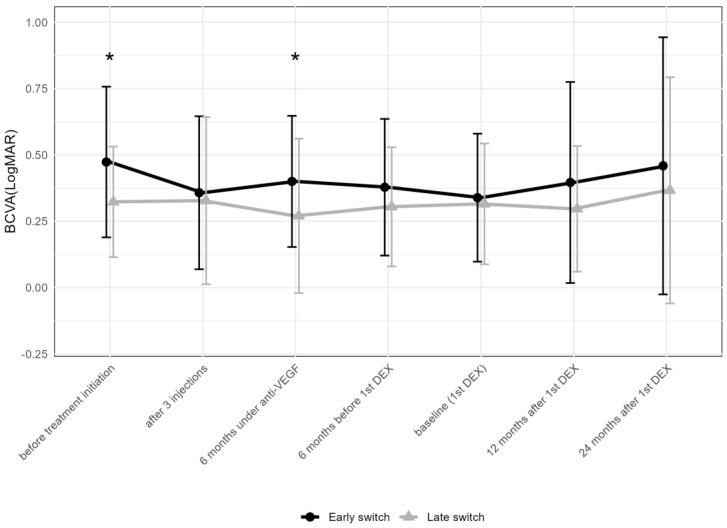
Comparison of best-corrected visual acuity between early (within 6–26 months after the first anti-VEGF injection) and late (within 27–106 months) addition of DEX. The data points are shown as mean and standard deviation (whiskers). * *p* < 0.05 by the Mann–Whitney U test; DEX = intravitreal dexamethasone implant; anti-VEGF = anti-vascular endothelial growth factor.

To compare the change in BCVA following the early and late addition of DEX, patients were divided into two groups based on the median time of the first DEX of 27 months. In the “early” group (addition of DEX within 6–26 months after the first anti-VEGF injection), BCVA in logMAR improved from 0.2 ± 0.8 (median 0.4; IQR: 0.1–0.7) at the initiation of anti-VEGF treatment to 0 ± 0.8 (median 0.2; IQR: 0.1–0.5) after three anti-VEGF injections (Figure 5), remaining stable thereafter: 0.1 ± 0.8 (median 0.2; IQR: 0.1–0.5) at the first DEX injection, 0.1 ± 0.8 (median 0.2; IQR: 0–0.5) at 12 months, and 0.1 ± 0.7 (median 0.2; IQR: 0.1–0.4) at 24 months after the first DEX. In contrast, in the “late” group (addition of DEX within 27–106 months), BCVA in logMAR changed from 0 ± 0.8 (median 0.3; IQR: 0.1–0.4) to 0 ± 0.8 (median 0.2; IQR: 0–0.3) after three anti-VEGF injections, remaining stable thereafter: 0 ± 0.8 (median 0.2; IQR: 0.1–0.4) at the first DEX, 0 ± 0.8 (median 0.2; IQR: 0–0.4) at 12 months, and 0 ± 0.8 (median 0.2; IQR: 0–0.3) at 24 months after the first DEX.

When comparing the early vs. late addition of DEX regarding BCVA, the two groups showed a difference before treatment initiation (*p* = 0.039), but not at the addition of DEX or later. DEX was obviously added despite a satisfying functional response to anti-VEGF drugs in a majority of eyes. In both groups, BCVA remained stable after the first DEX (Figure 5). When comparing the number of total DEX between the eyes receiving the early and late addition of DEX, eyes with an early addition received less DEX compared to the late group (4.3 ± 4.1 compared to 7.3 ± 6.4, Mann–Whitney U test: *p* = 0.024). When comparing the number of anti-VEGF injections in the first and second year after the first DEX, no difference between the two groups was found.

### 2.6. Timing of the First DEX: Short (6–12 Months) vs. Long (>36 Months) 

**Figure 6:**
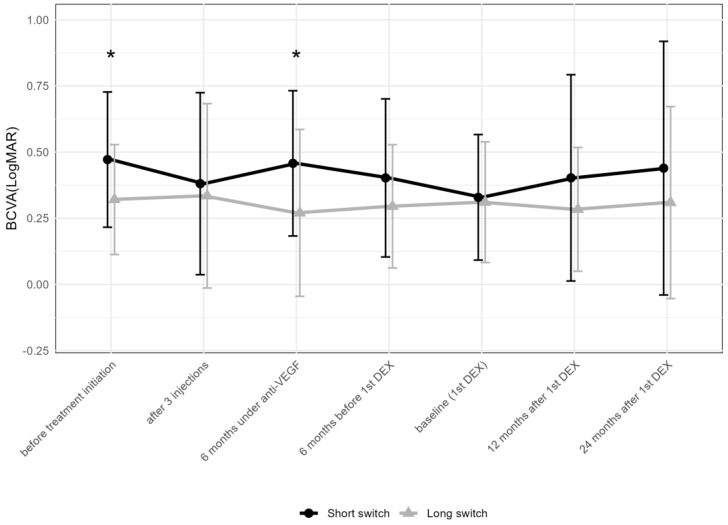
Comparison of best-corrected visual acuity between the groups receiving the first DEX after short-term (6–12 months) vs. long-term (over 36 months) anti-VEGF treatment. The data points are shown as mean and standard deviation (whiskers). * *p* < 0.05 by the Mann–Whitney U test between the “short” and “long” groups; DEX: intravitreal dexamethasone implant; anti-VEGF = anti-vascular endothelial growth factor.

There were 23 and 38 eyes meeting the criteria for the “short” and “long” anti-VEGF treatment time prior to the first DEX, respectively. BCVA at initiation (*p* = 0.018) and 6 months after the introduction of anti-VEGF therapy (*p* = 0.012) differed between the two groups (Figure 6), but not thereafter.

While BCVA improved from the initiation of anti-VEGF treatment to the first DEX in the “short” group and remained stable thereafter, it remained stable at all time points compared to baseline in the “long” group. 

**Figure 7:**
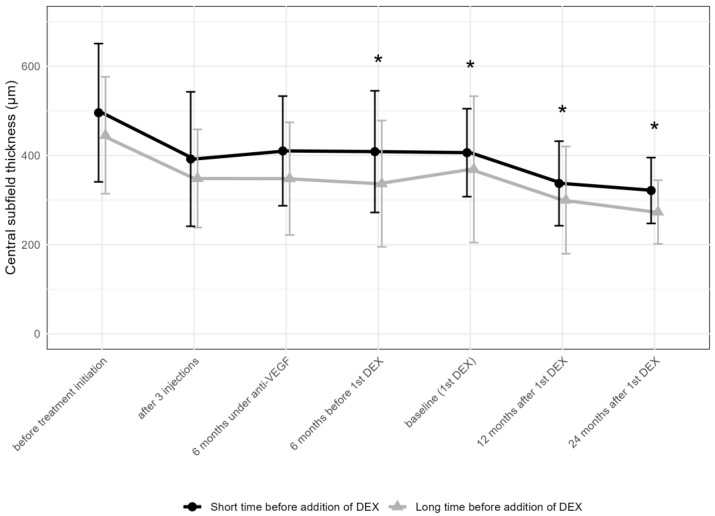
Comparison of central subfield thickness between the groups receiving the first DEX after short-term (6–12 months) vs. long-term (over 36 months) anti-VEGF treatment. * *p* < 0.05 on Mann–Whitney U test between the “short” and “long” groups; DEX: intravitreal dexamethasone implant.

The thickness of the CST decreased in both groups significantly after the addition of DEX. In the “short” group, this change was less pronounced, while the “long” group achieved a greater reduction in CST (Figure 7).

### 2.7. Adverse Events 

During the study period, 25 eyes (25.3%) needed new or additional intraocular pressure (IOP)-lowering agents after DEX, glaucoma surgery was required in 4 eyes (4.2%), and cataract surgery was performed in 1 eye six months following the first DEX. 

## 3. Discussion 

This retrospective multicenter study conducted across Swiss health care centers demonstrates that independent of the time gap between the initiation of anti-VEGF treatment and the addition of DEX, most eyes with DME achieve a relevant anatomic improvement. This goes along with a stabilization of BCVA in nearly 50% and a two-line improvement in BCVA in 14.7% of subjects over two years, as well as a relevantly reduced treatment burden over at least two years. The fact that the addition of DEX did not result in more significant functional improvements cannot be attributed to the timing of its administration, namely the late consideration of DEX. We rather understand this as evidence that in real life, clinical decisions represent a compromise between optimizing the long-term morphologic and functional outcomes and supporting patient adherence by including individual needs in the treatment decision. The absence of differences in our results between early and late DEX addition may indicate that clinical decision-making balances these conflicting interests. It does so by using all therapeutic options, including switch and combination therapies between anti-VEGF and DEX, to maintain a supportable visual function. Baseline BCVA was lower in early DEX users, who responded well to anti-VEGF drugs regarding anatomic and functional outcomes, so that BCVA and CST were similar to those in late DEX users. After addition or switch, however, they received less DEX than the late group, indicating treatment burden as an argument for the addition of DEX in these cases. In contrast to neovascular age-related macular degeneration, the aim of retinal fluid absence does not seem to be achievable in real life in all patients with DME. 

Our findings reconfirm that based on their strong anti-inflammatory properties, corticosteroids have significant efficacy in drying the retina even in anti-VEGF pre-treated eyes with a high treatment demand, making them encouraging options for DME patients who do not achieve adequate control of retinal fluid with anti-VEGF therapy alone [4,7,13]. 

### 3.1. Stabilization of Visual Acuity and Disease Control

VA holds paramount importance in the management of DME, as it is directly correlated with patients’ quality of life [14]. Already in previous studies, the switch to DEX in patients with chronic or refractory DME resulted in an improvement in VA and reduction in CST; however, the visual gains were not always sustained [15,16,17,18]. Other studies did not show similar gains, and thus, some controversy remains in the field [19]. When added to a monthly intravitreal therapy with ranibizumab, no significant visual benefit was encountered over 24 months in the DRCR Retina Protocol U study; however, the DEX combination group had a larger decrease in retinal thickness and a larger proportion of DME resolution [20], which is well in line with our experience. Furthermore, a recent systematic review and meta-analysis conducted in non-resistant DME found no differences between DEX and anti-VEGF monotherapies; however, in cases of resistant DME, the analysis revealed a greater improvement in BCVA with the use of DEX [21]. DEX therapy, moreover, resulted in greater CST reductions in both groups of DEX and anti-VEGF monotherapy [21]. 

Within our patient cohort, the incorporation of DEX alongside anti-VEGF therapy led to the stabilization of BCVA over two years. While we did not observe a net visual gain, the introduction of DEX may have prevented deterioration in visual function, given a mean CST of 380 μm at the addition of DEX. Following the first DEX treatment, a sustained 15% reduction in CST and absence of fluid in almost every third eye was registered at both 12 and 24 months, underscoring its impact on long-term functional stability, which is consistent with existing literature [22,23]. Comparing our CST data (mean of 480 μm) at the initiation of anti-VEGF to pivotal trials of ranibizumab and aflibercept (VISTA, VIVID, RISE, RIDE) suggests a comparable initial disease severity [24,25]. Notably, DEX therapy, particularly in cases of persistent macular edema despite anti-VEGF therapy, plays a central role in preventing further visual impairment [26]. It may achieve this by averting complications such as the disorganization of retinal inner layers (DRIL), ellipsoid zone disintegration, and photoreceptor loss, thus preserving long-term visual integrity [27].

### 3.2. Timing of the First DEX: Early Versus Late

The timing of the first DEX injection in our study is unique compared to published evidence in that the addition of DEX was considered after a minimum of six months under anti-VEGF therapy, with an average of 2.5 years and 16 anti-VEGF injections. The prolonged course of anti-VEGF therapy despite a high treatment demand from an early stage in a majority of these patients raises questions about the optimal time for adding DEX therapy. In a recent study in six eyes of four patients having “super-refractory” DME, that is, with a minimum of 15 injections, a CST over 380 μm, and failure to improve vision, the injection of DEX resulted in an improvement in vision and a reduction in CST [28]. Interestingly, the mean initial CST in our series was similar to this value, and 38 patients had more than 15 anti-VEGF injections prior to the addition of DEX. The frequency of DEX was reduced in the early administration group compared to the late administration group (4.3 ± 4.1 versus 7.3 ± 6.4). That anatomic and functional outcomes were similar despite a lower amount of DEX supports clinical evidence that long-standing edema does respond less rapidly, resulting in a higher treatment demand.

Our study, marked by a delayed first DEX in the majority of eyes after an average of 2.5 years into anti-VEGF treatment, indicates that even the late addition of DEX may well be beneficial for the patient, although better functional results could possibly be achieved if complete absence of fluid were achieved early, as in a minority of our cases. 

### 3.3. Injection Frequency and Safety

Before DEX therapy, our patients underwent an average of 16 anti-VEGF injections over 2.5 years or 1 injection every 8 weeks. Following the addition of DEX, the frequency of injections decreased substantially. This reduction in treatment frequency carries several noteworthy implications: 1. Reduced treatment burden may contribute to a better treatment adherence in DME patients, who often struggle with treatment compliance due to factors such as being of working age and the necessity of frequent medical appointments [29,30]. 2. Fewer injections lead to reduced direct medical expenses related to drug acquisition, administration, and monitoring [15]. Both factors may be especially crucial for the long-term management of DME, wherein consistent treatment is vital.

Though our study is not sufficiently powered to contribute relevant safety signals, the safety assessment of DEX therapy showed a well-supportable safety profile consistent with previous reports. Approximately one-quarter of participants needed IOP-lowering therapy, and four individuals underwent IOP-lowering surgery. The fact that only one participant needed cataract surgery may be associated with the initially high number of pseudophakic patients at the time of the first DEX.

### 3.4. Limitations

Despite the clinical relevance of our findings, our cohort study has several limitations. As a retrospective analysis, it may inherently suffer from inconsistencies, missing data due to documentation errors, and potential biases resulting from the lack of a standard protocol. In contrast to clinical studies, comorbidities, patient compliance with appointments, patient wishes, and center-specific treatment preferences are of major impact and a relevant limiting factor for long-term outcomes, which adds to the value of DEX in the armamentarium for the treatment of patients with DME. To mitigate these issues, we meticulously defined our assessment criteria during the study design. Additionally, the variable timing of DEX therapy during the observed period may introduce some variability.

Moreover, our baseline data indicate that patients receiving DEX had remarkably elevated baseline CST and relatively poor VA, suggesting a negative pre-selection of eyes with an advanced disease stage at the commencement of anti-VEGF treatment, which could have affected the prognosis. Furthermore, we were not able to assess disease severity at baseline due to the absence of widefield imaging and angiographic data, which could introduce a further bias. However, our study was observational in nature, with no intent to assess switching practices among Swiss retina specialists.

It is also important to consider the reduced injection frequency in patients with a prolonged treatment-free period before DEX administration. These eyes likely did not experience aggressive rebound or persistent edema, resulting in fewer injections after transitioning to either anti-VEGF or DEX therapy. This suggests that not all eyes in our cohort should be classified under the “insufficient anti-VEGF response” category, particularly those with extended intervals between treatments.

## 4. Methods

This retrospective multicenter cohort study was conducted at seven specialized eye centers in Switzerland, including Stadtspital Triemli, Zurich; Berner Augenklinik, Bern; Pallas Kliniken, Olten; VISTA Augenklinik, Binningen; Jules Gonin University Hospital, Lausanne; Swiss Visio Montchoisi, Lausanne; and Centre Ophtalmologique de la Colline, Geneve. This study received approval from the Ethics Committee of Canton Bern (registration number 2022-00510) and obtained general consent from all participating patients to utilize their coded data for this retrospective analysis. The study was conducted in accordance with the guidelines outlined in the International Council for Harmonization E6 Good Clinical Practice Guideline, the Declaration of Helsinki, and federal laws.

### 4.1. Subjects

Patients with DME who underwent a minimum of six months of pretreatment with intravitreal anti-VEGF agents, treated according to a treat-and-extend regimen and receiving three loading-phase injections of anti-VEGF drugs, prior to switching to or receiving adjunct DEX therapy between 2012 and 2021 were included. Exclusion criteria were the following: Snellen BCVA below 0.1 (corresponding to 1.0 logMAR), structural damage to the macula without functional potential, systemic co-morbidities that could interfere with treatment outcomes (such as local or systemic rheumatological diseases and/or vasculitis requiring anti-inflammatory treatment), any previous retinal therapies other than anti-VEGF (such as photodynamic therapy, macular laser or radiotherapy, vitreoretinal surgery), or triamcinolone injections within the last six months prior to the first DEX, presence of other underlying ocular diseases (including end-stage glaucoma, uveitis, vitreoretinal traction, and/or tractional epiretinal membrane), aphakia, and complicated cataract surgery within six months prior to the first DEX, as well as refusal to provide general consent.

### 4.2. Application of the Intravitreal Dexamethason Implant

Patients in the study were treated with a biodegradable intravitreal dexamethasone implant (Ozurdex®; Allergan, an AbbVie company, North Chicago, IL, USA). Each implant contains 0.7 mg of dexamethasone and is designed to provide a sustained release of the drug for up to six months.

### 4.3. Data Collection

Data were retrospectively collected from electronic patient records prior to therapy initiation with anti-VEGF, at 3 and 6 months of anti-VEGF therapy, 6 months prior to the first DEX, at the time of the addition of DEX (baseline), and 12 and 24 months thereafter. The following parameters were extracted: BCVA measured on a Snellen decimal scale and converted to logMAR for the statistical assessments, IOP, CST, as well as the presence of intra- and subretinal fluid on optical coherence tomography (OCT; Spectralis OCT, Heidelberg Engineering, Heidelberg, Germany in all participating centers), as judged by the investigators. The last treatment interval before the first DEX, as well as the number and dates of intravitreal injections per year after the first DEX, were also recorded. Based on the chart review, the reasons for the addition of DEX were recorded as “persistent fluid”, “high treatment demand”, and “treatment burden not supported by the patient”.

In order to compare the change in BCVA and CST following the early and late addition of DEX therapy, patients were divided into two groups of “early” and “late” DEX users, grouped compared to the median time of the first DEX. Furthermore, we also created two subgroups of the early and late addition of DEX, being added 6–12 months or over 36 months following the first anti-VEGF therapy (“short” and “long” DEX users, respectively). 

### 4.4. Statistical Analysis

Descriptive statistics were applied to present the demographic data. The distribution pattern of the data was assessed using the Kolmogorov–Smirnov test, which indicated that the data did not follow a normal distribution. The data are presented as mean ± standard deviation (SD) and, where indicated, as median with interquartile range (IQR) representing the 25th and 75th percentiles. For the above reasons, Wilcoxon signed-rank test was used for the comparison of paired data across different time points. The Friedman test, a non-parametric alternative to the one-way repeated measurement ANOVA, was used to compare multiple time points. Mann–Whitney U and Kruskal–Wallis H tests were used to compare subgroups. Statistical analyses were conducted using SPSS software package V.27 (SPSS, Inc., Chicago, IL, USA) and R (version 3.2.4; R: A language and environment for statistical computing, R Foundation for Statistical Computing, Vienna, Austria, 2016) with a level of significance at *p* < 0.05.

## 5. Conclusions

Our multicenter retrospective study demonstrates the benefits of DEX therapy in the management of insufficiently controlled DME with a high treatment demand. After the addition of DEX, our patients witnessed a significant reduction in CST, indicating improved disease control, as well as functional stability. Our findings underscore the value of reduced treatment frequency achievable through the incorporation of DEX therapy. Furthermore, they highlight the generally cautious approach taken by Swiss clinicians when considering the integration of DEX therapy into the treatment regimen for DME. These results contribute to the ongoing discourse on the optimal management of DME, emphasizing the multifaceted approach required to address this complex and vision-threatening condition. They also emphasize the need for further research with long-term follow-up to refine treatment strategies, i.e., the earlier addition of DEX in patients insufficiently responsive to or compliant with anti-VEGF drugs in order to enhance the long-term outcomes in DME.

## Figures and Tables

**Table 1:** Number of intravitreal anti-VEGF injections administered in the 12 months prior to the first DEX and in the two years thereafter.

	N	Mean ± SD (Min–Max)	IQR 25%	75%	*p*-Value
Year prior to first DEX	95	6.4 ± 3.1 (0.8–12.4)	4.1	9.1	
After first DEX	95 (1st year)	1.6 ± 2.4 (0–10.0)	0.0	3.0	*p* < 0.001
	89 (2nd year)	2.1 ± 2.8 (0–12.0)	0.0	4.0	*p* < 0.001

DEX: intravitreal dexamethasone implant; anti-VEGF: anti-vascular endothelial growth factor; SD: standard deviation; IQR: interquartile range.

**Table 2:** Time until the first DEX implantation and its correlation with visual acuity.

	At First DEX	12 Months after First DEX	24 Months after First DEX
N	95	87	70
Pearson’s Correlation	0.08 ^1^	0.14 ^1^	0.11 ^1^
*p*-Value	0.47	0.21	0.36

^1^ There were no correlations at any of the time point after the first intravitreal dexamethasone (DEX) implant injection.

**Table 3:** Course of central subfield thickness from the first anti-VEGF treatment to the last follow-up at 24 months after the first DEX.

	N	Mean ± SD (Min–Max)	Median	IQR		*p*-Value *
				25%	75%	
Prior to first anti-VEGF	90	477.7 ± 141.0 (174.0–799.0)	473.5	356.0	591.8	
After 3 anti-VEGF	78	366.0 ± 120.4 (144.0–775.0)	337.5	272.5	440.3	
At 1st DEX	95	378.1 ± 130.4 (146.0–910.3)	354.0	290.0	446.0	
6 months after 1st DEX	88	317.8 ± 109.1 (154.0–697.0)	291.5	244.3	350.0	<0.001
12 months after 1st DEX	88	327.3 ± 121.5 (163.0–712.0)	299.0	248.0	381.8	<0.001
24 months after 1st DEX	71	320.4 ± 125.5 (112.0–771.0)	282.0	244.0	363.0	0.006

DEX: intravitreal dexamethasone implant; IQR: interquartile range; SD: standard deviation. * *p*-Values indicate the results of the Friedman test compared to the first DEX.

**Table 4:** Course of central subfield thickness from first anti-VEGF treatment to the last follow-up in the three subgroups.

	Total Sample	Multiple DEX Implants + Anti-VEGF (*n* = 42)	Single DEX Implant + Anti-VEGF (*n* = 24)	DEX Monotherapy (*n* = 21)	*p* Values *
Prior to 1st anti-VEGF	477.7 ± 141.0 Median 473.5 (356.0–591.8)	482.7 ± 141.6 Median 474.0 (393.0–576.0)	431.0 ± 144.0 Median 404.0 (305.0–522.0)	536.0 ± 127.5 Median 537.5 (437.0–642.6)	0.07
After 3 anti-VEGF	366.0 ± 120.4 Median 337.5 (272.5–440.3)	384.2 ± 140.3 Median 373.0 (273.0–447.8)	329.7 ± 86.0 Median 292.0 (262.8–408.8)	374.0 ± 93.9 Median 359.0 (298.5–440.3)	0.41
At 1st DEX	378.1 ± 130.4 Median 354.0 (290.0–446.0)	379.3 ± 151.5 Median 357.0 (276.8–459.3)	360.1 ± 92.2 Median 345.5 (292.8–427.5)	401.0 ± 129.6 Median 362.0 (322.5–485.0)	0.77
12 months after 1st DEX	327.3 ± 121.5 Median 299.0 (248.0–381.8)	327.4 ± 133.7 Median 310.0 (215.0–396.0)	326.3 ± 103.0 Median 295.5 (262.8–353.8)	320.5 ± 107.4 Median 304.0 (249.5–338.3)	0.99
24 months after 1st DEX	320.4 ± 125.5 Median 282.0 (244.0–363.0)	292.6 ± 108.7 Median 277.0 (225.0–335.0)	337.6 ± 130.8 Median 285.5 (260.5–371.5)	360.0 ± 145.6 Median 324.0 (244.5–453.8)	0.15

Eight eyes with only one intravitreal dexamethasone (DEX) implant implantation and no further treatment, thereafter, are not reported in this table. DEX: intravitreal dexamethasone implant; SD: standard deviation. * Kruskal–Wallis H test.

## Data Availability

Data is contained within the article.
